# Betel Quid and Oral Potentially Malignant Disorders in a Periurban Township in Myanmar

**DOI:** 10.1371/journal.pone.0162081

**Published:** 2016-09-09

**Authors:** Ko-Ko Zaw, Mya Ohnmar, Moh-Moh Hlaing, Yin-Thet-Nu Oo, Swe-Swe Win, Maung-Maung-Than Htike, Phyu-Phyu Aye, Sein Shwe, Moe-Thida Htwe, Zaw-Moe Thein

**Affiliations:** 1 Department of Medical Research (Lower Myanmar), Yangon, Myanmar; 2 University of Dental Medicine, Yangon, Myanmar; 3 International Health Division, Ministry of Health, Nayphyitaw, Myanmar; 4 Department of Public Health, Nayphyitaw, Myanmar; Navodaya Dental College and Hospital, INDIA

## Abstract

This study aims to describe betel quid chewing practice and compare oral potentially malignant disorders between chewers and non-chewers of betel quid among residents in Dagon Myothit (East) Township, Myanmar. The study used a cross-sectional design conducted with a representative sample of 542 adults aged 18 years and above in the township. The trained interviewers collected data using a pretested structured questionnaire. On-site oral examination was done for suspected oral lesions. The mean age of the respondents was 45 years and 59% were women. Fifty-two percent of the respondents were currently in the habit of chewing betel quids (72% of men and 39% of women). Among 284 current betel quid chewers, 240 (85%) chewed betel quids together with tobacco. Out of 284 current betel quid chewers, 24 (8.5%) were found to have oral potentially malignant disorders; out of 258 betel quid non-chewers, only 1 (0.4%) was found to have oral potentially malignant disorders. This highlights the growing importance of smokeless tobacco use as public health problem.

## Introduction

An estimated 600 million people worldwide, 10% of the world’s population, chew betel quid [1]. Betel quid chewing has been common in South and Southeast Asia, Asia Pacific Region including Myanmar for a long time [2]. Its prevalence varies from 5% in Karachi, Pakistan through 49% in Sarawak, Indonesia up to 80% in parts of India [1,3]. Betel quid chewing is an ancient practice in Myanmar dating back to Takaung Historical Period and prevalent since Pagan Period in Myanmar and interwoven into social customs and religious practices [4].

Betel quid basically contains areca nut, slaked lime and catechu, wrapped in betel leaf, with tobacco commonly added [3]. The basic ingredients may be supplemented with spices (such as cloves, cardamom and aniseed) sweetening agents (such as coconut) as per individual preference.

Betel quid use has multiple impact on health [5]. Areca nut, the primary ingredient in betel quid, form nitrosamines in the saliva of chewers which induces oral preneoplastic disorders with a high propensity to progress to cancer [1]. Areca nut, with or without added tobacco, is an established cause of oral cancer, pharyngeal and esophageal cancers [1,5,6,7]. Areca nut with added tobacco is recently found to cause pancreatic tumours [7]. There is also limited evidence that areca nut causes liver cancer [1]. Recent epidemiological studies have shown that betel quid use is associated with a higher risk of obesity, metabolic syndromes, cardiovascular disease, type 2 diabetes and chronic kidney disease, low birth weight and cirrhosis of the liver [8].

Betel quid chewing is commonly associated with smokeless tobacco use, which impose additional health risks to users. In Myanmar, 30% of the adult population was using smokeless tobacco in 2009 and the majority of these smokeless tobacco users used it in a form of betel quid [9]. Because of anti-smoking initiatives in the country, prevalence of smoking is getting lower (22% in 2009) but that of smokeless tobacco use (30%), mostly in betel quid, is still high [9]. It seems that great social acceptance of smokeless tobacco use contributes to high prevalence of smokeless tobacco use in Myanmar [9].

This study investigates the details of betel quid chewing practice in a selected township. The finding from this study is expected to contribute to the database useful for designing the anti-tobacco intervention specially targeting the smokeless tobacco use and the oral cancer prevention and control measures.

This study aims to describe knowledge about and practice of betel quid chewing and compare prevalence of Oral Potentially Malignant Disorders between betel quid chewer and non-chewers in Dagon Myothit (East) township. The specific objectives of the study are: to determine the prevalence of betel quid chewing in adult population of Dagon Myothit (East) township; to describe the characteristics of betel quid chewing practice; and to compare oral pre-cancerous lesions between chewers and non-chewers of betel quid.

## Methodology

The study used a cross-sectional design and was conducted in Dagon Myothit (East) township, Yangon Region. The study population was persons aged 18 years and above of both sexes but excluded institutionalized people (armed forces, hospitalized patients and monks), very ill persons and mentally ill persons.

The sample size is calculated using the formula for one sample proportion with the following assumptions: alpha error is set at 5%, so ‘*z’* is 1.96; P was conservatively estimated at 50% because the exact prevalence of betel quid chewing in Myanmar is unknown; margin of error (e) is set at 5%; rate of refusal to participate was assumed to be 10%. The cluster sampling design was used with the design effect set at 1.5. The required sample size was determined to be 428 persons and the actual total sample size recruited into the study was 542 persons. From Dagon Myo Thit (East) Township, 2 enumeration areas were randomly selected. The household list of selected enumeration areas were requested form the local authority. From the household list of selected enumeration areas, five hundred and forty two households were selected at random. From each selected household, one eligible person was selected. If a selected household had more than one eligible person, only eligible person was selected at random from the list of eligible persons in this household.

By using a pretested structured questionnaire, the trained interviewers collected from the respondents the following data on:

1. socioeconomic characteristics
2. betel quid chewing practice
3. characteristics of betel quid chewing practice
  - age at which the respondent started betel quid chewing
  - duration of the practice
  - frequency of consumption of betel quid
  - constituents of betel quid (areca nut, betel leaves, slaked lime, catechu, tobacco, spices, essences, sweeteners, etc.)
  - chewing habits (spit out or shallow the juice)
4. alcohol drinking
5. smoking

The dental surgeons made oral examination on every respondent and applied Toluidine blue and oral brush biopsy on the respondents with visible oral lesions. When these oral lesions were positive by both Toluidine blue stain and oral brush biopsy, these were regarded as potentially malignant.

Data from the questionnaires were entered into the computer by the research assistants at DMR using data checking system with Epi Data program. Prevalence of betel quid chewing was described by age and sex and 95% confidence intervals were given for men and women and both sexes. Characteristics of betel quid chewing practice were described for men and women and both sexes. Prevalence of oral potentially malignant disorders was compared between betel quid users and non-user. Multiple logistic regression was performed to determine the independent effect of each of age, sex and the major habitual life styles on Oral Potentially Malignant Disorders, controlling for possible confounders.

Initially, 5 variables (age, sex, betel chewing, smoking and alcohol drinking), all of which were defined as categorical, were tested with log-likelihood test for inclusion in the model. In the final model, only 4 variables (age, sex, betel chewing and alcohol drinking) remained: smoking was omitted because it was multicollinear with alcohol drinking.

The proposal was approved for ethical clearance by the Ethical Review Committee of the Department of Medical Research. Written informed consent was obtained by interviewers from the respondents for face-to-face interview and oral examination by inspection and Toluidine blue stain and oral brush biopsy. The persons who tested positive for Toluidine blue stain and oral brush biopsy were referred to University of Dental Medicine for further investigation, regular follow-up and necessary treatment.

## Results

### Background characteristics

The study involved interview and oral examination of 542 persons aged 18 year and above in Dagon Myothit (East) township in 2013. Nearly half of the study population was 45 years and older. Women made up about 60% of the study population.

### Prevalence of betel quid chewing habit

Table 1 shows the prevalence of current betel quid chewing (without tobacco, with tobacco, and overall) among the respondents by sex and age groups. The overall prevalence of current betel quid chewing was 52% (95% confidence interval = 48%-57%), with prevalence in men much higher than that in women (72% vs. 39%). Out of 284 current betel quid chewers, 240 (85%) chewed betel quids with one kind of tobacco or another (not shown in the table). The highest prevalence current betel quid chewing was found in 24–44 years age group.

**Table 1:** Prevalence of betel quid chewing among the respondents by sex and age.

		Chewers of betel quid		
Sex and age	Pop.	without tobacco	with tobacco	total
***Male***	***221***	***7 [3*.*2%]***	***153 [69*.*2%]***	***160 [72*.*4%]***
		***[95%CI = 1*.*2 to 6*.*4%]***	***[95%CI = 62*.*7 to75*.*2%]***	***[95%CI = 66 to 78*.*2%]***
18–24 years	28	2 [7.1%]	19 [67.9%]	21 [75.0%]
24–44 years	92	1 [1.1%]	74 [80.4%]	75 [81.5%]
45–64 years	78	4 [5.1%]	49 [62.8%]	53 [68.0%]
65+ years	23	0 [0.0%]	11 [47.8%]	11 [48.8%]
***Female***	***321***	***37 [11*.*5%]***	***87 [27*.*1%]***	***124 [38*.*6%]***
		***[95%CI = 8*.*2 to 15*.*5%]***	***[95%CI = 22*.*3 to 32*.*3%]***	***[95%CI = 33*.*3 to 4*.*2%]***
18–24 years	31	2 [6.5%]	2 [6.5%]	4 [12.9%]
24–44 years	128	13 [10.2%]	38 [29.7%]	51 [39.8%]
45–64 years	125	17 [13.6%]	41 [32.8%]	58 [46.4%]
65+ years	37	5 [13.5%]	6 [16.2%]	11 [29.7%]
***Total***	***542***	***44 [8*.*1%]***	***240 [44*.*3%]***	***284 [52*.*4%]***
		***[95%CI = 6 to10*.*7%]***	***[95%CI = 40 to 48*.*6%]***	***[95%CI = 48 to 56*.*7%]***
18–24 years	59	4 [6.8%]	21 [35.6%]	25 [42.4%]
24–44 years	200	14 [6.4%]	112 [10.9%]	126 [57.3%]
45–64 years	203	21 [10.3%]	90 [44.3%]	111 [54.7%]
65+ years	60	5 [8.3%]	17 [28.3%]	22 [36.7%]

CI = confidence interval

### Kinds of tobacco consumed with betel quids among chewers betel quid with tobacco

Table 2 shows different kinds of tobacco consumed with betel quids among betel chewers by sex and age groups. The commonest accompanying ingredient was untreated tobacco leaves followed by “92” brand of tobacco leaves which is imported from India.

**Table 2:** Kinds of tobacco consumed with betel quids among chewers betel quid with tobacco.

Type of tobacco	Male	Female	Total
	(n = 153)	(n = 87)	(n = 240)
Tobacco leaves (untreated)	99 [64.7%]	52 [59.8%]	151 [62.9%]
“92” tobacco	59 [38.6%]	15 [17.2%]	74 [30.8%]
Tobacco leaves (tenderized)	30 [19.6%]	17 [19.5%]	47 [19.6%]
“Parajet” tobacco	27 [17.7%]	10 [11.5%]	37 [15.4%]
Tobacco leaves (steamed)	12 [7.8%]	6 [6.9%]	18 [7.5%]
“45” tobacco	12 [7.8%]	5 [5.8%]	17 [7.1%]
“100” tobacco	8 [5.2%]	2 [2.3%]	10 [4.2%]
“Signal” tobacco	6 [3.9%]	2 [2.3%]	8 [3.3%]
“Star” tobacco	2 [1.3%]	3 [3.5%]	5 [2.1%]

### Characteristics of betel quid chewing

Table 3 shows characteristics of betel quid chewing practice among the respondents who currently chewed betel quids. There were 284 current betel quid chewers (160 were men and 124 were women). Among them, half started chewing betel quids at 25 years or earlier, half chewed betel quids for 10 years or longer and half chewed 8 betel quids or more per days. Most of current betel quid chewers spitted out betel quid juice but about one in ten of them swallowed it sometimes or always. The commonest way of discarding used betel quid among the betel chewers was spitting out it onto the ground or building corner or whatever place available at the time. The main reasons given for chewing betel quids is ‘to ease an sour sensation in the mouth’ (35%) and ‘addiction to chewing betel quids’ (35%).

**Table 3:** Characteristics of betel quid chewing practice among the respondents who currently chewed betel quids.

	Male	Female	Total
	(n = 160)	(n = 124)	(n = 284)
Median age of starting betel quid chewing [Range]	22 years [10–61]	32 years [10–60]	25 years [10–61]
Median duration of betel quid chewing [Range]	11.0 years [0–57]	8.5 years [0–75]	10.0 years [0–75]
Median number of betel quids per day [Range]	10 quids [1–100]	6 quids [1–50]	8 quids [1–100]
Way of dealing with betel quid			
*-Swallow*	5 [3.1%]	3 [2.4%]	8 [2.8%]
*-Spit out*	139 [86.9%]	113 [91.1%]	252 [88.7%]
*-Both Swallow and Spit out*	16 [10%]	8 [6.5%]	24 [8.5%]
Way of discarding used betel quid			
*-Spit out onto the ground/building corner*	116 [72.5%]	78 [63.9%]	194 [68.8%]
*- Spit out to plastic bags or other containers*	41 [25.6%]	46 [37.7%]	87 [30.9%]
-*Spit out to dustbin/dump*	16 [10.0%]	3 [2.5%	19 [6.7%]
Reason for chewing betel quid*			
*- To ease an sour sensation in the mouth*	59 [36.8]	40 [32.8%]	99 [35.1%]
*- Because of addiction to chewing betel quids*	55 [34.4%]	42 [34.4%]	97 [34.4%]
*-To be alert*	35 [21.9%]	6 [4.9%]	41 [14.5%]
*-To be concentrated*	21 [13.1%]	7 [5.7%]	28 [9.9%]
*-To make breaths sweet*	13 [8.1%]	14 [11.5%]	27 [9.6%]
*-To quit smoking*	13 [8.1%]	1 [0.8%]	14 [5.0%]

*Multiple responses

### Relationship between major lifestyles and Oral Potentially Malignant Disorders

Table 4 shows prevalence and unadjusted risk of Oral Potentially Malignant Disorders by major lifestyles. Out of 542 persons, 25 persons (4.6%) turned out to have visible oral lesions (ulcer or patch) which tested positive for Toludine blue staining and oral brush biopsy (95%CI = 3.0 to 6.7). These were regarded as potentially malignant.

**Table 4:** Prevalence and unadjusted risk of Oral Potentially Malignant Disorders by major lifestyles.

	Pop.	Oral Potentially Malignant Disorders		
		Number	Percent [95% CI]	Crude Odds Ratio
**Betel quid chewing habit**				
*Non-chewers (Ref*. *group)*	258	1	0.4 [0 to 2.1]	1
*Chewers without tobacco*	44	1	2.3 [0.06 to 12]	6 [2 to 17]
*Chewers with tobacco*	240	23	9.6 [6.2 to 14]	27 [12 to 62]
**Smoking habit**				
*Non-smoker (Ref*. *group)*	371	17	4.6 [2.7 to 7.2]	1
*Smoker*	171	8	4.7 [2.0 to 9.0]	1.02 [0.4 to 2.6]
**Alcohol drinking habit**				
*Non-drinker (Ref*. *group)*	451	17	3.8 [2.2 to 6.0]	1
*Drinker*	91	8	8.8 [3.9 to 16.6]	2.5 [1.1 to 5.7]

CI = Confidence interval

The prevalence of potentially malignant oral lesions rose from non-chewers through chewers without tobacco to chewers without tobacco. The prevalence of potentially malignant oral lesions was similar between non-smokers and smokers. The prevalence of potentially malignant oral lesions was significantly higher in alcohol drinkers than in non-drinkers.

Crude odds ratios show that betel hewers were 6 times more likely to have Oral Potentially Malignant Disorders than non-chewers and that risk rises to 27 times with addition of smokeless tobacco to betel quid. Drinkers were nearly 3 times more likely to have Oral Potentially Malignant Disorders than non-drinkers.

Table 5 shows the adjusted odds ratios of Oral Potentially Malignant Disorders for age, sex, betel chewing and alcohol drinking from the final multiple logistic regression model. As shown in Table 4 betel chewing are significantly associated with risk of Oral Potentially Malignant Disorders. Alcohol drinking, and female are also associated with risk of Oral Potentially Malignant Disorders but their relationship was not significant.

**Table 5:** Odds ratios of Oral Potentially Malignant Disorders from multiple logistic regression according to demographic and major lifestyles characteristics.

	Adjusted Odds Ratio	95% Confidence Interval
**Age**		
*< = 40 years (Ref*. *group)*	1	
*40–60 years*	1.5	0.6 to 4.2
*60+ years*	2.0	0.7 to 5.7
**Sex**		
*Female (Ref*. *group)*	1	
*Male*	0.7	0.3 to 1.7
**Betel quid chewing habit**		
*Non-chewers (Ref*. *group)*	1	
*Chewers without tobacco*	5.7	1.4 to 22.9
*Chewers with tobacco*	28.6	9.8 to 83.6
**Alcohol drinking habit**		
*Non-drinker (Ref*. *group)*	1	
*Drinker*	1.6	0.7 to 4.0

CI = Confidence interval

## Discussion

The survey provided a profile of betel chewing practice in adult population living in a periurban area of Yangon Region. Half of this population were currently chewing betel quids and This prevalence of betel chewing is quite high given the globally estimated prevalence of 10–20% [2] and prevalence in some Asian countries like 20%-40% prevalence found in India, Pakistan and Nepal over the last two decades [10,8], 9.8% in Malaysia, 10.7% in Thaiwan, 12% in Indonesia, 18% in Sri Lanka, 23.9% in mainland China [8] and 31% in Bangladesh [11].

Adding tobacco with betel quid is a common practice in the South-East Asian countries. In the current study, 85% of the betel quid chewers added tobacco which is higher than 35.5% for Sri Lanka [8], 63.6% for Malaysia [8], 70.4% for Indonesia [8], 85.2% for Dhaka, Bangladesh by Rahman et al [12]. This high rate of adding tobacco to betel chewing in this study is also in sharp contrast with Thaiwan and mainland China where there is practically no habit of adding tobacco to betel chewing [8].

Practice of current betel chewing was more prevalent in men (72%) than in women (39%). This pattern of male preponderance in betel chewing is also found in many Asian countries like Sri Lanka, Nepal, Thaiwan and mainland China but female preponderance in betel chewing was found in Malaysia and Indonesia [8]. Extent of current betel quid chewing was highest (57%) in 24–44 years age group of the respondents as a whole but highest (46%) in 45–64 years age group among the women.

Betel quid chewers mostly started betel chewing practice around 25 years of age, chewed 8 betel quids per day for 10 years or more. Most betel quid chewers spitted out betel quid juice Betel quid chewers usually discarded used betel quid onto the ground or building corner or whatever place available at the time. They chewed betel quids mainly because they want to ease sour sensation in the mouth’ and they were addicted to betel chewing.

A small fraction of this study population (4.6%) had potentially malignant oral lesions as determined by rapid screening tests (Toludine blue staining and oral brush biopsy) and these potentially malignant oral lesions were almost exclusively confined to current betel quid chewers. Prevalence of potentially malignant oral lesions in current chewers of betel quid without tobacco (2.3%) increased to nearly 10% with addition of smokeless tobacco to betel quid. This finding provides additional evidence for formulating policy on control of smokeless tobacco use in Myanmar.

Toluidine blue staining and oral brush biopsy are found to be are useful diagnostic aids in community setting but they, especially toluidine blue staining, have some limitations in diagnostic accuracy [13, 14]. Oral brush biopsy showed high specificity and sensitivity in detection of oral potentially malignant disordersand toluidine blue staining was highly sensitive and moderately specific in high-risk populations and suspicious mucosal lesions but less sensitive for premalignant lesions [13,14]. Therefore clinicians and researcher should refer the people with abnormal results of toluidine blue staining and brush biopsy to specialty referral and/or tissue biopsy for definitive diagnosis of oral potentially malignant disorders and necessary treatment [14].

Multiple logistic regression indicated that older age, betel chewing, especially with tobacco and consumption of alcohol were associated with risk of Oral Potentially Malignant Disorders. These findings are consistent with the internationally established risk factors for risk of Oral Potentially Malignant Disorders [5].

In conclusion, betel quid chewing was found to be a common habit in both men and women of the study population. Because betel quid chewing have serious health consequences an anti-betel quid chewing programme is warranted for current chewers. Education about betel quid chewing should be emphasized in the public prevention education. Regular screening for betel quid chewing may help prevent avoidable oral cancers in the future. As the habit is rooted in Myanmar tradition and culture, anthropological studies are indicated for designing appropriate educational campaigns.

## Supporting Information

S1 Dataset (DTA)
